# Horizontal Gene Transfer among Bacteria and Its Role in Biological Evolution

**DOI:** 10.3390/life4020217

**Published:** 2014-05-16

**Authors:** Werner Arber

**Affiliations:** Biozentrum, University of Basel, Klingelbergstr. 50/70, CH-4056 Basel, Switzerland; E-Mail: Werner.Arber@unibas.ch; Tel: +41-61-267-2130; Fax: +41-61-267-2118

**Keywords:** microbial genetics, gene vectors, recombinant DNA molecules, genetic variation, gene acquisition, evolution genes, natural selection, microbiome, gene domestication, laws of nature

## Abstract

This is a contribution to the history of scientific advance in the past 70 years concerning the identification of genetic information, its molecular structure, the identification of its functions and the molecular mechanisms of its evolution. Particular attention is thereby given to horizontal gene transfer among microorganisms, as well as to biosafety considerations with regard to beneficial applications of acquired scientific knowledge.

## 1. Introduction

The discovery of horizontal gene transfer is related to the introduction of experimental microbial genetics some 70 years ago. Soon thereafter, medical microbiology identified the raising problem of increasing antibiotic resistance of pathogenic bacteria. This medical problem stimulated research in bacterial genetics which revealed that horizontal gene transfer is involved in some of the genetic variations causing resistance to antibiotics.

As a contribution to the history of scientific investigations, we trace here a sequence of steps of conceptual and experimental approaches to understand microbial evolution at the molecular level. This shall allow us to extrapolate to generally valid laws of nature guiding biological evolution by self-organization. We will also discuss implications of the acquired scientific knowledge.

## 2. Roots of Microbial Genetics

It is in the first half of the 20th century that microbiologists became aware that bacterial isolates and bacterial viruses (bacteriophages) under study could spontaneously produce phenotypic variants. This property offered the chance to investigate recombination between different mutants and between different microbial strains, *i.e.*, to carry out genetic experiments with bacteria as well as with bacteriophages.

### 2.1. The Discovery of Bacterial Transformation

Early work with *pneumococcal* bacteria has identified two forms: smooth (S) cells which are virulent, and rough (R) cells which are not virulent as seen upon infection of mice in laboratory tests. Using this experimental system, Griffith [1] infected mice both with living R variants and with heat-killed S bacteria. After some time, many of the doubly infected mice developed pneumonia and in their blood S type bacteria were found. This indicated that recombination had occurred between the living R form and a substance of the heat-killed S form bacteria. In the subsequent search for the identity of the substance causing virulence, Avery, MacLeod and McCarty [2] identified a highly purified DNA fraction to bring about recombinants, whereas no other fraction of the heat-killed S bacteria caused recombination. This rather unexpected result clearly indicated that DNA is the carrier of genetic information. A majority of biologists hesitated to accept this novel knowledge since one had expected that the highly complex and specific genetic information would rather be carried on more complex molecules than DNA, e.g., by proteins. This problem found its solution almost ten years later when Hershey and Chase [3] showed that bacteriophage T2 injects its DNA, but not proteins upon infection of host bacteria, and when the double-helical filamentous structure of DNA molecules was described by Watson and Crick [4]. These authors proposed genetic information to be contained in the specific sequences of nucleotides.

### 2.2. Bacterial Conjugation

In the same time period, several researchers also tested the possibility of recombination between bacterial isolates with different phenotypes upon mixed incubation. This happened to occur in some of the mixtures, in particular those involving phenotypic mutants of certain *Escherichia coli* strains [5]. This phenomenon involving a tight contact between the donor cell and the recipient cell was called conjugation. In further experimental investigations conjugation could be seen to depend on the presence in the donor cell of a so-called fertility factor F, a relatively small autonomous DNA molecule [6]. Occasionally, F integrates into the bacterial chromosome to produce Hfr derivatives. Both in the autonomous and in the integrated state, F can give rise to pairs with F^−^ recipient bacteria. This allows a copy of the replicating F to enter into the partner cell. From its integrated Hfr state, the F transfer normally also transfers neighboring parts of the donor chromosome. This kind of experiments led to the conclusion that the *E. coli* genome is one large circular DNA molecule. Occasionally, F undergoes unprecise excision from the Hfr chromosome. It thereby becomes autonomous again and carries in addition to its own genes some genes from its bacterial host. Such hybrid derivatives are called F’ and they are actually vectors for bacterial genes which can become transferred into recipient bacteria upon conjugation (see [6,7]).

F’-like conjugative plasmids have soon become known to act sometimes in the horizontal transfer of antibiotic resistance determinants. This can seriously contribute to the spreading of these determinants to pathogenic bacteria, in particular in the presence of antibiotics exerting a selective pressure.

### 2.3. Bacteriophage-Mediated Transduction

Transduction was first seen in studies on gene exchange between *Salmonella* bacteria [8]. In later investigations it became clear that several different kinds of bacterial viruses can occasionally also serve as natural vectors for genes of their host bacteria (see [7]). Two different strategies were identified to contribute to this horizontal transfer of genes between different host bacteria. In generalized transduction, some of the propagating progeny viral particles contain a segment from the host chromosome rather than a reproduced viral genome. This is, for example, seen for the *Salmonella* phage P22 and for the *E. coli* phage P1. In contrast, in specialized transduction, the transducing viral particle contains a hybrid molecule with a part of the phage genes and some bacterial genes. In the late 1950’s I had the chance to identify for the first time such a hybrid genome in λgal derivatives of phage λ. This hybrid DNA molecule was still able to undergo autonomous replication, but instead of some of its viral coat genes, it carried genetic determinants for the fermentation of galactose picked up in the host chromosome [9]. Its full reproduction depended therefore on the presence of an intact “helper” phage genome.

## 3. Bacterial Restriction/Modification Systems Limit Horizontal Gene Transfer

Upon genetic experimentation using several bacterial strains and their phages, a phenomenon called host-controlled modification was encountered by a few independent scientists. In the 1960’s we succeeded to unravel its molecular mechanisms [10]. In brief, many strains of bacteria possess one or even more than one genetic set-ups to identify invading foreign DNA as foreign and to start its destruction by endonucleolytic cleavage (restriction enzymes). If the invader is a bacteriophage, its DNA can escape restriction with a quite low probability. The viral genome becomes thereby insensitive to restriction by site-specific methylation (modification). This is a kind of epigenetic alteration that does not affect the genetic information carried in the phage genome. Reproduced modified phage then grows well without restriction in its new host, but often not any longer in its previous host. Restriction acts upon phage infection as well as upon transformation and conjugation between two bacterial strains carrying different restriction/modification capacities. We conclude that bacterial restriction/modification systems drastically limit successful horizontal gene transfer in the world of bacteria. This contributes to the genetic stability, but it still allows occasional genetic variation by horizontal gene transfer to occur as a contribution to biological evolution.

## 4. Other Natural Barriers against Functionally Relevant Horizontal Gene Transfer

In this context, it might be relevant to briefly mention here that in the world of bacteria and their viruses, several other barriers are known to limit the rates of uptake of foreign genetic information. Among these are a lack of bacterial surface compatibility for conjugational pair formation or for the uptake of DNA molecules from the medium, bacteriophage host range specificities, a requirement for functional harmony between the invading genetic information with the recipient’s genome, and a rather unexpected microbial immunity, called CRISPR-Cas, acquired upon a previous infection [11].

## 5. *In Vitro* Recombinant DNA Molecules and Their Use in Fundamental and in Applied Genetic Research

In view of the large size of DNA molecules carrying the genomic information of any organism, researchers reflected on possibilities to sort out particular genes and to multiply them in view of their structural and functional studies. Viral DNA molecules and F conjugative plasmids were envisaged to serve as autonomously replicating gene vectors, into which a relatively short DNA fragment could be spliced. Such hybrids might then serve for studies of both structural and functional properties of the inserted DNA fragment. This possibility became realistic when bacterial restriction enzymes became available around 1970 [12].

Scientists involved in this work discussed measures to be taken in order to prevent undesirable effects of recombinant DNA molecules with yet unknown genetic information. Such conjectural effects might affect the health of the investigator, on the one hand, and they might also render possible an undesirable spreading of the inserted genetic information to other organisms after an accidental or a deliberate release into the environment, on the other hand [13]. The answer to the latter concern required a better understanding of the laws of nature guiding spontaneously occurring genetic variation, the driving force of biological evolution.

## 6. Molecular Mechanisms and Natural Strategies of Genetic Variation

Both, already available data on spontaneous genetic variation and novel experimental results, in particular from microbial genetics, revealed a multitude of specific molecular mechanisms to contribute to the overall spontaneous genetic variation. We have classified these identified molecular mechanisms of genetic variation into three natural strategies of genetic variation [14]. These are: (a) Local alterations of nucleotide sequences, such as a nucleotide substitution, the insertion or the deletion of one or a few adjacent nucleotides. Such spontaneously occurring variations are often linked with DNA replication; (b) A second strategy of nature to produce genetic variations resides in a duplication, deletion, inversion or translocation of a DNA segment carried in the genome; (c) The third strategy of genetic variation consists in the acquisition by horizontal gene transfer of a segment of foreign DNA. Transformation, conjugation and viral infection can contribute to these effects. It is clear that the maintenance of any novel genetic variant is depending on Darwinian natural selection.

These natural laws of genetic variation are solidly shown to apply to microorganisms. Fewer experimental data are at this time available for higher organisms, but reports on cross-species gene transfer [15], as well as recent DNA sequence comparisons, speak clearly in favor of a general validity of the relevant natural laws of genetic variation for all living organisms.

### 6.1. Conceptual Implications of the Observed Laws of Genetic Variations

Experimental observations show that in spontaneously occurring genetic variation specific gene products are often involved, on the one hand, and/or non-genetic properties of the non-living world, on the other hand. Some of the involved gene products act as variation generators, others as modulators of the rates of genetic variation, which provides to the living organisms a relatively high genetic stability required for maintenance of life [14]. Many of the gene products required for a relatively slow but steady biological evolution are not essential for individual lives from one generation to the next. We therefore label their genetic information as evolution genes. As a philosophical consequence we note a dual nature of the genome. Many genes serve the individual life, while the evolution genes principally serve for the biological evolution of the population, a requirement for adaptation to changing habitats upon the terrestrial evolution of our planet [16].

In this self-organized biological evolution nature uses, besides the activities of evolution genes, a number of already mentioned non-genetic elements such as structural flexibilities (isomeric forms) and limited chemical stability of biological molecules, random encounter and environmental mutagens (chemicals and radiations). Random encounter is of particular relevance for horizontal gene transfer.

### 6.2. Implications of Horizontal Gene Transfer

The natural strategy to occasionally accept foreign genetic information by horizontal gene transfer links all living organisms into a global system. Whereas Darwin had drawn the evolutionary tree linking different organisms at the bottom of the tree, we can now, in addition, interlink different branches of the tree with horizontal connectors in order to allow for horizontal transfer of individual genes [17]. Being aware that horizontal gene transfer will also contribute to future events of genetic variation, we can conclude that the preservation of the encountered high biodiversity with a rich diversity of genetic information is essential and a guarantee for a harmonious long-term biological evolution on our planet. Note that a common language, *i.e.*, the universality of the genetic code, plays thereby an essential role [15,18].

### 6.3. Cohabitation Favors Horizontal Gene Transfer

In recent years it became obvious that all kinds of higher organisms, plants, animals and humans carry in and on their bodies a high number of microorganisms. These are only rarely pathogenic. They rather profit from a stable symbiosis. The higher organisms offer to bacteria good habitats, and bacteria contribute in a number of ways (food digestion, cleaning up of the skin, *etc.*) to a healthy life of their hosts. This cohabitation is called microbiome and it can be maintained for long periods of time, whereby the microorganisms reside sometimes outside and in other cases inside of the cells of their host organism. Any such cohabitation must favor an occasional gene transfer between the involved organisms [18]. Such gene transfer can occur in both directions.

### 6.4. Evolutionary Inventiveness of Nature’s Self-Organization

Our present scientific insights into the evolution of life and of appropriate habitats reveal a remarkable degree of invention concerning different specific molecular processes that contribute to the generation of a rich diversity of forms of life living in a large number of different habitats. It becomes more and more known that many specific molecular mechanisms can contribute to a slow but steady evolutionary progress of living organisms. We have become aware that mechanistic insights into specific steps of genetic variation obtained in the work with one kind of bacteria cannot be generalized for all kinds of bacteria. On the other hand, it seems to us that the three defined natural strategies of genetic variation (local sequence change, intragenomic rearrangement of DNA segments, horizontal gene transfer) can best contribute to conceptually understand the self-organized natural process of the slow but steady evolution of life to a rich biodiversity within a global system of interdependencies. This does not only concern the genomic information, but also compositional differences of appropriate habitats, including the availability of healthy nutrition.

### 6.5. Time-Scale of Evolutionary Processes

In our daily life we are used to observe and to understand relatively fast processes. However, both the evolution of life and cosmic evolution are extremely slow, long-term processes which we have difficulty to perceive with our sensory organs. This may be a possible reason why a number of people (including some life scientists) still remain anchored in a fundamentalistic world-view claiming that there is no evolution. From an updated scientific view-point, we understand that a fast evolution of life could be detrimental. We assume that in the long past of biological evolution, the evolution gene activities of variation generators and of modulators of the rates of genetic variation have become fine-tuned to work as they do today. Note that spontaneous genetic variation is contingent so that by far not all genetic variants show improved functions [19]. Therefore, a rapid evolutionary progress would rather lead to an eradication of forms of life than to a steady contribution to enrich the treasure of biodiversity.

### 6.6. The Natural Strategies of Genetic Variation Contribute to Biological Evolution with Different Qualities

Let us start with horizontal gene transfer, *i.e.*, the acquisition of genetic information that had been developed in other kinds of living organisms. By chance, a gene acquisition can provide to the receiving organism a welcome novel function that provides a selective advantage. An example is the acquisition of genetic information for antibiotic resistance by pathogenic bacteria. We see here a sharing in the evolutionary success of other organisms. Of course, horizontal gene transfer can, in other cases, also lead to a selective disadvantage by negative impacts on the functional harmony of the receiving organism.

The quality of local mutagenesis resides mainly in a possible stepwise improvement of an already existing biological function. Again, such mutations are contingent and not precisely targeted as a response to identified needs.

Intragenomic DNA rearrangements offer possibilities to fuse functional segments of DNA sequences; this can occasionally result in novel activities. The fusion between two different functional domains may, by a rare chance, lead to a novel, welcome function. Or the fusion of a functional gene with an alternative control element for gene expression can influence the expression of the concerned gene.

We have to be aware that in all these events, functional improvements can be a relatively rare consequence, while a selective disadvantage and in the extreme case lethality are more likely to result. The self-organizing biological evolution requires thereby a sacrifice from one individual in a large population undergoing a slow but steady evolution. Let us mention in this context that religious persons may encounter conceptual difficulties to accept that genetic adaptation to novel life conditions occurs with some contingency.

## 7. The Role of Horizontal Gene Transfer in Synthetic Biology

As we have already outlined above, in the recombinant DNA technique selected DNA sequences are sorted out and spliced into other DNA molecules such as natural gene vectors. This and other procedures, such as site-directed mutagenesis, have become important contributions to the so-called synthetic biology that can serve for useful applications of fundamental scientific knowledge on biological functions. Insights into principles of natural strategies of genetic variation as outlined in Section 6 can thereby help to prevent conjectural risks of experimentation and of its products [20]. A general advice of relevance is the recommendation that natural laws of genetic variation as discussed in Section 6, should be respected in the *in vitro* construction of genomic segments. With regard to the formation of recombinant DNA molecules, this precautionary principle can validly contribute to the biosafety of the envisaged product of biosynthesis. Interesting examples of *in vitro* transfer of useful genetic information into another living organism allowing for a domesticated production and harvest of the relevant gene product were reported recently [21]. We can predict that future investigations will continue to offer welcome availabilities of identified genes and of their products which can serve for beneficial use to the service of humankind and of its environment. It is likely that horizontal gene transfer will thereby continue to play a major role.

## Supplementary Files

Supplementary File 1 Review Report (PDF, 137 KB)
