# Supplementary material for: Horizontal Gene Transfer among Bacteria and Its Role in Biological Evolution
Source: Life (Basel). 2014 May 16;4(2):217–24. doi: 10.3390/life4020217 (PMC4187160; doi:10.3390/life4020217)
Supplement: Supplementary File 1 — Review Report (PDF, 137 KB) [file life-04-00217-s001.pdf]

ISSN 2075-1729
www.mdpi.com/journal/life

Peer-Review Record:

Horizontal Gene Transfer among Bacteria and Its Role in Biological Evolution

Werner Arber

***Life* 2014, *4*, 217-224, doi:10.3390/life4020217**

Reviewer 1: Anonymous
Reviewer 2: Anonymous
Reviewer 3: Anonymous
Editor: Pabulo Henrique Rampelotto (Federal University of Pampa, Brazil, *Editor-in-Chief* of *Life*)

Received: 19 February 2014
First Editorial Decision: 20 March 2014
First Revision Received: 22 April 2014
Accepted: 23 April 2014
Published: 16 May 2014

First Round of Evaluation

Round 1: Reviewer 1 Report

This article seems most appropriate for an issue of Life devoted to "Horizontal Gene Transfer and the Last Universal Common Ancestor". The manuscript provides a clear overview of the various mechanisms employed by microorganisms for horizontal gene transfer and their contribution to the evolutionary process. The author is a highly respected authority in the field. Still, I have a couple of minor suggestions/comments:

- Section 2.1: I think it would be fair to refer to the work of Hershey and Chase, because it is my impression that their experiments with radioactively labeled phages were decisive to confirm DNA as the carrier of genetic information, as previously proposed by Avery *et al.*;
- In addition to restriction/modification systems, there is another system that immunizes bacteria and archea against invading genetic elements, namely, the CRIPR-Cas system. Since it is highly likely that it has also played an important role in evolution, it would be worthwhile to mention it, perhaps in Section 3. A recent review on the subject can be found in *Cell. Mol. Life Sci.* **2014**, *71*, 449–465;

- It is not clear what the author means by “and/or non-genetic properties of the non-living world” in Section 5.1. Perhaps the author could be more specific and mention some of these properties;
- The author could also be more specific when he states that “A general advice of relevance is the recommendation that natural laws of genetic variation should be respected in the *in vitro* construction of genomic segment”. (for example?)

Round 1: Author Response to Reviewer 1

All four suggestions were followed by introducing additional references to publications or to specific examples.

Round 1: Reviewer 2 Report

The manuscript life-51396 submitted by Werner Arber is a very short review about the role of horizontal gene transfer in biological evolution and the historical roots of gene transfer knowledge in the foundational works of the molecular biology.

General Comments

In my opinion, this work encompasses two different parts. The first one tracks the historical roots of horizontal gene transfer back to the seminal works in both bacterial transformation and conjugation and phage-mediated transduction and it links horizontal gene transfer knowledge to the development of molecular biology and genetic engineering as scientific disciplines. The second one discusses the horizontal gene transfer role (as a mechanism for acquisition of genetic variation), in the evolution of the life world.

I think that the manuscript length is very short to discuss in depth all the information that the proposed topic deserves:

1. The first part of the manuscript is, in my opinion, the most original and most interesting. It tracks the origins of horizontal gene transfer back to the early years of the molecular biology and shows how the understanding of transformation, conjugation and transduction drives development of genetic engineering and the conceptual implication that genes can be transferred between different organisms. In addition, it discusses how the bacteria restriction and modification systems are efficient barriers to horizontal transfer of genetic material.

   Despite being acknowledged in several papers that mechanisms of horizontal gene transfer have been known since the origins of molecular biology, a discussion of horizontal gene transfer links to molecular biology development as proposed by the author is welcome.

   However, at these early times, implications for horizontal gene transfer as an evolutionary mechanism were ignored (in part because the microbes have been out of the Modern Synthesis that permeated the evolutionary theory during most of the 20th century (see for example, Sapp and Fox (2013) The Singular Quest for a Universal Tree of Life. *Microbiol. Mol. Biol. Rev. 77*, 541–555) and it was not until the Syvanen paper (Syvanen, M. (1985) Cross-Species Gene Transfer: Implications for a New Theory of Evolution, *J. Theor. Biol. 112*, 333–343) that horizontal gene transfer gains a place in evolutionary thinking. Other papers appearing in the early 1990s—the

cited paper from Arber (1991) Elements in Microbial Evolution. *J. Mol. Evol. 33*, 4–12 or Hilario and Gogarten (1993) Horizontal Gene Transfer of ATPase Genes—The Tree of Life becomes a Net of Life. *Biosystems 31*, 111–119, for example—reinforce the role of horizontal gene transfer such as an evolutionary mechanism.

I think that this part of the manuscript should be extended and improved, discussing how horizontal gene transfer has been incorporated to evolutionary thinking following the development of genomic and post-genomic eras.

2. The other part of the manuscript discusses briefly the role of horizontal gene transfer in evolution of the life world. In my opinion, this part is very short in length and their contribution to abundant literature in this topic is small (several recent reviews in this topic have been published (Ragan *et al.* (2009) *Philos. Trans R. Soc. Lond B. 364*, 2241–2251; Boto (2010) *Proc. R. Soc. B 277*, 819–827; Syvanen (2012) *Annu. Rev. Genet 46*, 341–358 for example). I think that this part deserves major elaboration.

In addition, a better link between the two manuscript parts could be welcome. Author links concerns on prevention of undesirable effects of recombinant DNA technology to efforts to understand the laws of nature driving genetic variation, and in the last term, an interesting link between horizontal gene transfer and Asilomar Conference is suggested. However, beyond the Asilomar Conference, is the ulterior development of new and powerful tools for DNA study (that leads to the so called Genomic Era) and a new focus in microbes as evolutionary subjects that paved the way to place horizontal gene transfer in evolutionary thinking.

For these reasons, I should like to suggest to the author a review of the manuscript according to one of two possible options:

1. To focus the paper on the history of horizontal gene transfer discussing how horizontal gene transfer (whose origins are linked to the born and development of molecular biology) has been incorporated to evolutionary thinking in the last 25 years. If this option is chosen, I suggest changing the manuscript title according to the new scope.
2. To maintain the current scope of the paper extending both parts to encompass the emerging information accumulated in the last 25 years.

Particular Comments

1. At the end of Section 4, the author claims that genetic variation is the driving force of biological evolution. I think that the real driving force of evolution is natural selection operating in natural genetic variation.
2. In Section 5.1, the author distinguishes evolution genes from other genes relating to individual life of organisms. However, the differences between both types of genes are not clear from the manuscript. I think that this is a consequence of the short length of the manuscript and it would be interesting to improve this part in a new version of the manuscript.
3. In Section 5.3, the author discusses how cohabitation favors horizontal gene transfer. There are a lot of interesting recent references on this topic not cited or discussed in the manuscript (see for example Skippington, E.; Ragan, M.A. (2011) Lateral genetic transfer and the construction of

genetic exchange communities. *FEMS Microbiol. Rev 35*, 707–735; Bonn *et al.* (2014) Interactions in the Microbiome: Communities of Organisms and Communities of Genes. *FEMS Microbiol. Rev 38,* 90–118). I suggest extending this section to include relevant and recent bibliography addressing this topic.

4. Section 5.4: I suggest changing evolutionary progress to evolutionary change.
5. In Section 5.5, the author discusses the pace of evolutionary change claiming that because the evolution is a long-term process, it is difficult for human perception. However, *in vitro* microbial evolution studies such as that carried out by Lenski *et al.* (some with author collaboration, for example PNAS USA **1999**, *96*, 3807–3812) show that the perception of evolution is possible during the human lifetime. I think that this deserves to be clarified.
6. In the Section 5.6 I think that reference to difficulties to accept evolution by religious persons makes no sense.
7. Also in Section 5.6, the author introduces horizontal gene transfer as "the acquisition of genetic information that had been developed in other kinds of living organism". I think that introduction of horizontal gene transfer should be placed at the beginning of the paper

Round 1: Author Response to Reviewer 2

I did not consider to prepare an encyclopedic paper covering all aspects of horizontal gene transfer which could have resulted in an independent monograph. Rather, I felt that my task was to prepare a report on early investigations in microbial genetics and on the thereby obtained insights into the relevance of horizontal gene transfer for biological evolution and on underlying laws of nature. I still expect that other complementary articles will address important aspects, in particular regarding horizontal gene transfer in eukaryotes and also regarding future possibilities for practical applications such as by synthetic biology.

Particular points raised by referee 2:

1. Without any genetic variation in the genomes as the driving force of evolution, there would not be any biological evolution. Natural selection is not the driving force of evolution; it directs evolution into particular directions in cooperation with all spontaneously available variants and their parents.
2. In the clonal growth of a bacterial population, evolution genes are not essential for the duplication of an E. coli cell in only 30 minutes. Evolution genes, such as variation generators, act on very rare individuals in the population. The situation in eukaryotes with long generation times is more complex and some variation generators can also give rise to somatic mutations. But I generally abstained from including reference to eukaryotes in my article.
3. In the absence of proper information, I had assumed that another author would report in detail on aspects of the microbiome. I therefore mentioned cohabitation only briefly, in particular by referring to symbiosis, since the general public still looks at bacteria as its enemies.
4. I prefer "evolutionary progress" rather than "evolutionary change". This is a matter of world-view.
5. The general public does not look at rapidly growing bacterial populations. People look at their generation times and can hardly observe any sign of evolution.

6. I do not agree that reference made to religious persons improving conceptual difficulties to accept contingency of genetic variation is nonsense. In contrast, it is very important for us scientists to be aware of this difficulty to understand the laws of nature guiding biological evolution.
7. I cannot mention the different qualities of each natural strategy of genetic variation before having explained what these strategies are. Section 6.6 (new number) describes the principal qualitative differences between the three strategies which collectively contribute to a fruitfully functional biological evolution.

Round 1: Reviewer 3 Report

If this chapter is considered to be an introduction to the broader topic of horizontal gene transfer then something with this focus on the history is appropriate. It is well written and mostly accurate. But I do think that the author should acknowledge that the significance of transfer mechanisms he documents were not appreciated for many decades as I describe below.

This paper is entitled "Horizontal gene transfer and its role in biological evolution". Most of the paper describes the very relevant history of gene transfer mechanisms and other molecular genetic events controlling transfer as well as genetic engineering. This is a story that was put together between 1930 and 1970. In fact, 9 of the 16 references in this work were published before 1980. However, none of that work in its day was considered as mechanisms for horizontal gene transfer (HGT) as it is understood today; namely evolutionary significant transfers of genetic information between different species. Even after plasmid transfer between E. coli and Salmonella was discovered in 1955, the larger significance of gene transfer was largely over looked. Instead, those results were generally presented as bacterial sex as it was described in Stent's and Stanier's text books at the time. Even as late as 1978 a prominent paper appeared that claimed natural populations of *E. coli* were clonal. The role of HGT in biological evolution was not really addressed until the 1980s, though a few suggestions appeared in the 1970s. There has been a huge number of papers in the last two decades, especially in the last one, documenting cases of HGT (it is being found practically everywhere, plants, animals, protozoa and fungi besides bacteria). Obviously the literature is huge and cannot be covered in a single review. However, there have been changes in our thinking about biological evolution during this period that is mostly overlooked here.

A couple of points:

- Section 5.2: A point is made about the importance of a unified genetic code in facilitating horizontal gene transfer and a 2006 paper is cited. This argument first appeared in 1984 (as discussed in this recent review (Syvanen (2012). Evolutionary Implications of Horizontal Gene Transfer. *Annual Review of Genetics 46*, 341);
- Section 5.6: The role of tandem duplications as an important mechanism in the evolution of new functions is mentioned. The author seems to be unaware that HGT, and not tandem duplication, seems to be a more important source of duplicate homologs found in bacteria (Treangen T.J.; Rocha, E.P.C. (2011). HGT, Not Duplication, Drives the Expansion of Protein Families in Prokaryotes. *PLoS Genet 7*, e1001284).

Round 1: Author Response to Reviewer 3

I have briefly mentioned in Section 2.1, obstacles encountered in the acceptance of novel scientific insights into biological evolution by bioscientists. I am aware that even today some biological scientists do not accept the fundaments of molecular Darwinism. But I did not want to write a paper on communication difficulties, I rather felt it important to trace the paths leading to our, by now, solidly established scientific knowledge on biological evolution, to which horizontal gene transfer makes important contributions.

In my draft I had overlooked important contributions made by Syvanen. This gap has now been corrected.

Since tandem duplications and their role for what I call evolutionary trial and error processes have been widely discussed in textbooks, I felt that mention of a possible source by intragenomic DNA rearrangements would be appropriate. However, since I do not want to go into more details in my article, I now deleted the relevant sentence.

Second Round of Evaluation

Round 2: Reviewer 2 Report

The reviewed paper from Arber has been improved including a new section about barriers against horizontal gene transfer other than restriction/modification systems and a discussion of the role of non-genetic elements in the evolution of life.

However, my main concern remains: the paper does not fill the gap between the seminal works that revealed the possibility of horizontal gene transfer between microbes and the current acceptation of horizontal gene transfer as an evolutionary mechanism.

I understand that the aim of the author is not to write an encyclopedic paper, and I think that the proposed paper is an interesting report tracking back the origins of horizontal gene transfer to early investigations in microbiology, genetics and molecular biology. However, I also think that a brief discussion of how genomic approaches have contributed to the current acceptation of horizontal gene transfer as an evolutionary mechanism would be welcome and it could complete the interesting historical perspective that the paper proposes.

On the other hand, in relation to the author's answers to my particular points, some brief comments:

1. I agree with the author in that, without natural variation, natural selection makes no sense. For this reason, my statement said that "natural selection operating in natural variation" is the driving force of biological evolution. In any case I agree that discussion of this topic is out of the scope of the current paper.
2. I know the difference between evolution genes and other genes serving the individual lives of organisms. My concern is that, in my opinion, this difference is not clear to the reader considering the short length of this section. In any case I agree that cited references can lead the reader to a better understanding of this topic.
3. I understand the author's arguments, but a recent reference to microbial communities as gene exchange communities would be welcome.

4. Despite differences in world-views, I consider that the use of terms such as “evolutionary progress” can be misleading. I agree that “progress” is a property of the evolution as a process. But “progress” can also be interpreted as an outcome of the evolutionary process, suggesting a teleological view of evolution not sustained by the current knowledge on how evolution acts. It is in this sense that I think that terms such as “evolutionary progress” should be avoided or need to be qualified in a paper focused on evolution.
5. I think that studies of “*in vitro* microbial evolution” are very good examples of current evolution demonstrating that evolution is an observable phenomenon, and it could be interesting an introduction of these studies in the manuscript.
6. I think that it is important for us scientists to be aware of the difficulty to understand evolution both by religious and non-religious persons. For this reason I think that a particular reference to religious persons can be superfluous.
7. I agree with the author o n this point.

Round 2: Author Response to Reviewer 2

In my comment to the first revision of my paper, I made the editor aware that I intended to limit my contribution to prokaryotic microorganisms. I also made this goal visible by introducing “bacteria” into the title of the paper. I still consider that extending my paper into the eukaryotic world is not my task. In Section 6.4, I mention that even in the prokaryotic world, identified specific processes are not necessarily valid in other kinds of organisms. Nature is quite inventive and, if required, it will find ways to satisfy its needs. In today's view, what is true for *E.coli* is not necessarily true for other organisms, at least not with regard to specific identified molecular mechanisms.

Comments to particular points of the reviewer:

4. Webster defines “progress” as follows: a moving forward or onward; forward course; development; improvement; advance toward perfection or to a higher state.
   Synonyms are: progression, advance, advancement.
   These meanings correspond to what I intend to state by using the term “evolutionary progress”.
5. Only very few persons on our planet Earth work like Lenski and ourselves experimentally with organisms having very short generation times, which favors studies on specific molecular mechanisms of spontaneous genetic variation. Please look at Section 6 in my paper where results of such studies are presented in a condensed way. I do not want to go into details in my paper. For the interested reader, literature references are given.
6. I had made a short reference to obstacles in accepting scientific knowledge on genetic variation, particularly by some religious persons whose religious beliefs dominate over scientific views in their orientational knowledge. I had mentioned this difficulty in view of my personal experience in contacts with highly estimated persons. I do not want to go into more details in this paper, but I think that this situation also generally influences the rejection of evolution in a wide population of fundamentalists. To make my point of view more visible, I have now inserted “in particular” into line 224 (see revised manuscript).

© 2014 by the reviewers; licensee MDPI, Basel, Switzerland. This article is an open access article distributed under the terms and conditions of the Creative Commons Attribution license (http://creativecommons.org/licenses/by/3.0/).
